# Intra-host dynamics of mixed species malaria parasite infections in mice and mosquitoes

**DOI:** 10.1186/1475-2875-9-S2-O31

**Published:** 2010-10-20

**Authors:** Jianxia Tang, Megumi Inoue, Toshihiko Sunahara, Moe Kanda, Osamu Kaneko, Richard Culleton

**Affiliations:** 1 Department of Protozoology, Institute of Tropical Medicine (NEKKEN), Nagasaki University, 1-12-4 Sakamoto Nagasaki 852-852, Japan; 2 Department of International Health, Institute of Tropical Medicine (NEKKEN), Nagasaki, 1-12-4 Sakamoto Nagasaki 852-852, Japan

## Background

The distributions of human malaria parasite species overlap in most regions of the world where malaria is present, and co-infections involving two or more malaria parasites are common. Currently, very little is known about the consequences of any interactions that may occur between species during co-infection for disease severity and parasite transmission success. However, current anti-malarial interventions such as vector control and drug interventions and the future application of vaccines will and do have disproportionate effects on some species compared to others; with the ultimate consequence of reducing the number of species in circulation in any one area. We believe that such a situation warrants a clearer understanding of how the interactions between species affect malaria disease and transmission dynamics.

## Methods

As controlled competition experiments using human malaria parasites are currently practically impossible, we assessed the consequences of mixed-species infections on parasite fitness, disease severity and transmission success using the rodent malaria parasite species *Plasmodium chabaudi* (strains AS and CB), *P. yoelii yoelii* (CU) and *P. vinckei lentum* (DS). We compared the fitness of individual species within co-infections and in single species infections in mice. We also assessed the disease severity of single versus mixed infections in mice by measuring mortality rates, anaemia and weight loss. Finally, we compared the transmission success of parasites in single or mixed species infections by quantifying oocyst development in *Anopheles stephensi* mosquitoes.

## Results

**Figure 1:**
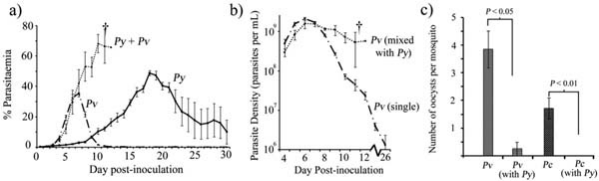
(a) Parasitaemia of mixed and single infections of *P.yoelii and P. vinckei,* in the mixed infection group, all mice died by day 12 post-inoculation. (b) Parasite density of *P. vinckei* in a mixed infection with *P.yoelii,* and in a single infection. (c) mean oocyst production per mosquito of *P*.vinckei and *P. chabaudi in* mixed infections with *P. yoelii,* and in a single infections. Data are means of five mice per group; error bars represent one standard error above and below the mean. *Pv = P. vinckei, Pc = P. chabaudi, Py = P.yoelii.* † = death.

We found that co-infections of *P. yoelii* with either *P. vinckei* or *P. chabaudi* led to a dramatic increase in infection virulence, with 100% mortality observed in mixed species infections, compared to no mortality for *P. yoelii* and *P. vinckei* single infections, and 40% mortality for *P. chabaudi* single infections. The increased mortality in the mixed infections was associated with an inability to clear parasitaemia (Figure 1a), with the non-*P*. *yoelii* parasite species persisting at higher parasite densities than in single infections (Figure 1b). *P. yoelii* growth was suppressed in all mixed infections compared to single infections. Transmissibility of *P. vinckei* and P. *chabaudi* to mosquitoes was also dramatically reduced in the presence of P. *yoelii* in co-infections compared to single infections (Figure 1c).

## Discussion

The increased virulence of co-infections containing *P. yoelii* (reticulocyte restricted) and P. *chabaudi* or P. *vinckei* (predominantly normocyte restricted) may be consequences of parasite cell tropism and/or immune modulation of the host. We explain the reduction in transmission success of species in co-infections in terms of inter-species gamete incompatibility.

